# PPAR*γ* and PPAR*δ* as Modulators of Neoplasia and Cell Fate

**DOI:** 10.1155/2008/247379

**Published:** 2008-06-12

**Authors:** Robert I. Glazer, Hongyan Yuan, Zhihui Xie, Yuzhi Yin

**Affiliations:** Department of Oncology and Lombardi Comprehensive Cancer Center, School of Medicine, Georgetown University, 3970 Reservoir Road, NW, Washington, DC 20007, USA

## Abstract

PPAR*γ* and PPAR*δ* agonists represent unique classes of drugs that act through their ability to modulate gene transcription associated with intermediary metabolism, differentiation, tumor suppression, and in some instances proliferation and cell adhesion. PPAR*γ* agonists are used by millions of people each year to treat type 2 diabetes but may also find additional utility as relatively nontoxic potentiators of chemotherapy. PPAR*δ* agonists produce complex actions as shown by their tumor promoting effects in rodents and their cholesterol-lowering action in dyslipidemias. There is now emerging evidence that PPARs regulate tumor suppressor genes and developmental pathways associated with transformation and cell fate determination. This review discusses the role of PPAR*γ* and PPAR*δ* agonists as modulators of these processes.

## 1. INTRODUCTION

PPAR*γ* and PPAR*δ* are involved in cell cycle regulation, survival and angiogenesis [1–3], and in inflammation through ligand-dependent and independent mechanisms [4]. Several recent reviews have described the role of PPARs in metabolic disease [4–6], cancer treatment [3, 7], and chemoprevention [8]. In addition to their metabolic actions, an emerging area of investigation for PPAR*γ* and PPAR*δ* agonists is their ability to modulate mammary cell lineage and genes associated with tumor suppressor function and cell fate determination. This suggests that PPAR agonists may play a role in stem/progenitor cell proliferation and differentiation to modify tumor response.

## 2. PPAR*γ* SIGNALING

The PPAR nuclear receptor subfamily consists of the PPAR*α*, PPAR*γ*, and PPAR*δ*/*β* isotypes that regulate a number of metabolic pathways controlling fatty acid *β*-oxidation, glucose utilization, cholesterol transport, energy balance, and adipocyte differentiation [4–6]. PPARs function as heterodimeric partners with RXR, and require high-affinity binding of PPAR ligand to engage transcription [7]. PPARs bind to the DR-1 response element (PPRE) consensus sequence AGG(T/A)CA, which is recognized specifically by the PPAR partner [9]. Like other nuclear receptors, PPARs consist of a putative N-terminal transactivation domain (AF-1), a DNA-binding domain (DBD) containing two zinc fingers, a ligand-binding domain (LBD) containing a large hydrophobic pocket, and a C-terminal ligand-dependent transactivation region (AF-2) [10].

There is >97% homology at the protein level, 99% homology within the LBD, and minimal functional differences after ligand-dependent activation between human and mouse PPAR*γ*, [11]. PPAR*γ* is expressed predominantly in white adipose tissue, intestine, endothelial cells, smooth muscle and macrophages [12], and is the major isotype expressed in the mammary gland, and in primary and metastatic breast cancer and breast cancer cell lines [3].

Several mutations and polymorphisms have been identified in PPAR*γ*, such as Lys319X (truncating) and Gln286Pro, in sporadic colon cancer, which are associated with loss of DNA-binding and ligand-dependent transcription by the PPAR*γ* agonist, troglitazone [13]. Similar results were found for PPAR*γ*2 polymorphism Pro112Ala [14], but the polymorphism Ser114Ala resulted in increased transactivation by presumably blocking the inhibitory effect of Ser114 phosphorylation by ERK [15, 16]. However, in a sampling of approximately 400 breast, prostate, colon, and lung tumors and leukemia's, no mutations of the PPAR*γ* gene were found, suggesting that if indeed this does occur, it is a very rare event [17].

**Figure 1:**
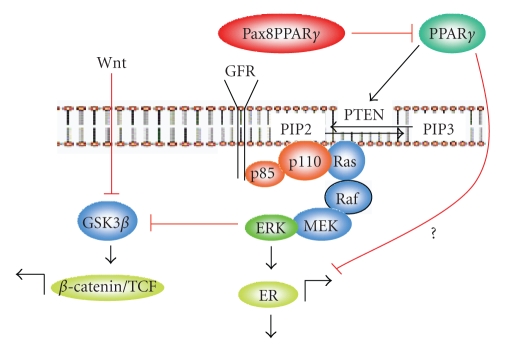
Pax8PPAR*γ* and mammary cell fate determination. Pax8PPAR*γ* acts in a dominant-negative fashion to block PPAR*γ*-dependent transactivation and upregulation of PTEN. MMTV-Pax8PPAR*γ* mice exhibit reduced PTEN and activation of Ras and ERK, presumably through activation of PI3K (*p85 and p110*). ERK activates ER transcriptionally and posttranslationally, and Pax8PPAR*γ* may interfere with the ability of PPAR*γ* to inhibit ER transactivation. Mammary epithelial cells isolated from the mammary glands of MMTV-Pax8PPAR*γ* mice contain a higher percentage of CD24^+^/CD29^hi^ stem/progenitor cells, and present with predominantly ER^+^ ductal carcinomas following carcinogenesis, suggesting a role of PPAR*γ* in cell fate determination.

In follicular thyroid cancer, the t(2;3)(q13;p25) translocation results in formation of the Pax8-PPAR*γ* fusion protein, which is pathoneumonic for the majority of cases of this disease [18]. It acts as a dominant-negative receptor of PPAR*γ* [18, 19], and reduces expression of the Ras tumor suppressor, NORE1A [20], which inhibits ERK activation [21]. PPAR*γ* also increases expression of other tumor suppressor genes, such as PTEN [22] and BRCA1 [23] through their respective PPRE promoter regions, suggesting that the antitumor effects of PPAR*γ* agonists may be related to their ability to downregulate multiple tumorigenic signaling pathways. This agrees with the reduction of PTEN and increased nuclear *β*-catenin and ERK activity in the mammary gland and tumors of MMTV-Pax8PPAR*γ* mice [24] (see Figure 1). Since inactivation of BRCA1 [25] and PTEN [26–28] also increases stem cell proliferation, Pax8-PPAR*γ* may upregulate specific progenitor cell lineages that are more susceptible to tumorigenesis.

PPARs interact with the coactivators C/EBP, SRC-1, and DRIP205, and in the unliganded state with the corepressor SMRT [19, 29–31], and exhibit similar coactivator/corepressor dynamics as other nuclear receptors, such as estrogen receptor-*α* (ER) [32]. PPAR*γ* can interfere with ER transactivation through its binding to the ERE [33, 34], and preferentially partitions with ER for its canonical response elements [35]; conversely, ER can block PPRE-dependent transcription [36] (see Figure 1). PPAR*γ* also modifies ER signaling by promoting its ubiquitination and degradation [37] as well as by upregulating CYP19A1 (aromatase) activity [38, 39], which can blunt the activity of aromatase inhibitors used to treat patients with ER^+^ breast cancer. PPAR*γ* agonists block the ER-dependent growth of leiomyoma cells, further suggesting crosstalk between the ER and PPAR*γ* signaling pathways. PPAR*γ* and ER pathways have opposite effects on PI3K/AKT signaling that may also account for the inhibitory action of PPAR*γ* ligands on ER-dependent breast cancer cells [36] (see Figure 1). These findings imply that PPAR*γ* antagonism should upregulate ER expression in responsive tissues, which is precisely the phenotype observed in mammary tumors induced in transgenic mice expressing Pax8PPAR*γ* [24].

Studies using transgenic and knockout mouse models of PPAR*γ* have led to disparate conclusions regarding the role of PPAR*γ* in tumorigenesis. Mice expressing constitutively active VP16-PPAR*γ* in the mammary gland did not exhibit a tumorigenic phenotype but accelerated tumorigenesis when crossed with MMTV-polyoma middle-T antigen mice [40], intimating that the unliganded receptor may have interfered with tumor suppressor transactivation by endogenous PPAR*γ* through corepressor recruitment. Alternatively, the VP16 fusion protein is known to induce many genes that are not indicative of PPAR*γ* activation [41]. In the probasin-SV40 T-antigen prostate tumor model, tumorigenesis was unaffected by a PPAR*γ* null background [42], indicating that oncogenic signaling was already maximally activated. However, in the Apc^Min^ mouse colon tumor model, “glitazone” PPAR*γ* agonists increased the number of colon, but not small intestine polyps [43, 44], as well as colon adenomas [45]. Since the small intestine, and not the colon, is the predominant site of neoplasia in this mouse model, the significance of this observation is unclear. It should also be stressed that PPAR*γ* agonists did not induce malignant changes in wild type mice, indicating their lack of carcinogenicity. Contrary to these results, PPAR*γ* haploinsufficiency produced a greater rate and number of colon tumors following azoxymethane-induced carcinogenesis [46], implying that PPAR*γ* acts as a tumor suppressor rather than as an oncogene. APC^+/1638N^ mice heterozygous for PPAR*γ* did not exhibit changes in polyp formation [46]. This result indicates that the induction of *β*-catenin in the colonic crypt cells of PPAR*γ* haplosufficient mice, a protumorigenic factor that is constitutively activated in APC mice, is the target of tumor suppression in wild-type mice [47]. A tumor suppressor role for PPAR*γ* is also supported by the inhibitory effect of PPAR*γ* agonists on colon tumor growth [48, 49], and mammary carcinogenesis [50–52]. This effect may be mediated in breast tumors through induction of apoptosis due to reduction of Bcl-2 [53], and in pancreatic and liver tumors through a reduction of cyclin D1 and HB-EGF [54] and an increase of p27^Kip1^ [55–57]. PPAR*γ* agonists may also find utility as modifiers of the response to chemotherapy. CS-7017, a potent thiazolidinedione agonist, synergized with paclitaxel to inhibit the growth of anaplastic thyroid tumors through induction of p21^Cip1^ [58]. Notwithstanding possible “off-target” effects [59, 60], most studies indicate that PPAR*γ* agonists as a class have antitumor activity, and thus may have efficacy as a relatively nontoxic adjunct to chemotherapy and possibly to radiation therapy through their ability to act as “tumor suppressor enhancers.”

## 3. PPAR*δ* SIGNALING

**Figure 2:**
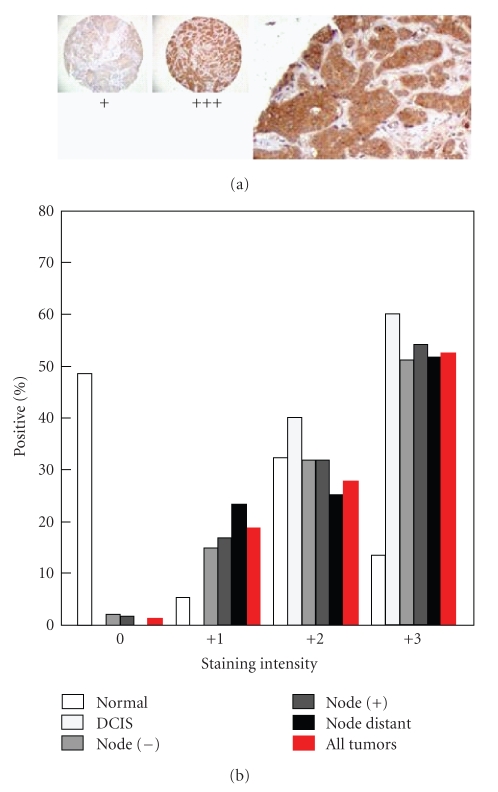
PPAR*δ* expression in invasive breast cancer. Representative samples from a tissue microarray analysis of invasive breast cancers are shown. PPAR*δ* staining intensity is indicated as low (+1), medium (+2) or high (+3). The magnified image shows examples of +1 and +3 staining. The bar graph depicts the percentage of samples expressing PPAR*δ* in DCIS, node (+), node (−) and node distant tumors.

As with PPAR*γ*, PPAR*δ* is involved in adipocyte differentiation by promoting clonal expansion of preadipocyte progenitor cells [61], possibly through activation of PPAR*γ* expression [62]. The PPAR*δ* agonist GW501516 has been tested clinically as a cholesterol lowering drug in dyslipidemic patients, but the results have been mixed [63]. In animal models, homozygous disruption of PPAR*δ* resulted in a runted phenotype [64] and in 90% embryonic lethality with runted survivors [65], indicating its importance in embryonic development. PPAR*δ* null macrophages exhibited loss of the dominant inhibitory effect by unliganded PPAR*δ* [60], which was previously identified by its ability to block PPAR*α* and PPAR*γ* transactivation through corepressor recruitment [60, 66, 67]. In breast cancer cells, PPAR*δ* expression was greater in ER^−^ MDA-MB-231 breast cancer cells than in ER^+^ MCF-7 cells [68], also suggesting a correlation with a more aggressive form of this disease. Indeed, tissue microarray analysis of invasive breast cancers indicated that PPAR*δ* is strongly expressed (see Figure 2, “+3”) in 52% of 164 samples, and thus may have value as a prognostic marker and therapeutic target. There are no examples of the development of PPAR*δ* *antagonists* as anticancer therapeutics.

GW501516 accelerated the onset of tumor formation during mammary carcinogenesis, in contrast to the delay of tumor formation by PPAR*γ* agonist GW7845 [52]. PPAR*δ* expression increased in K-Ras-transformed intestinal epithelial cells [69] and PDGF-stimulated vascular smooth muscle cells [70]. Similar findings were reported for conditional expression of PPAR*δ*, where GW501516 increased proliferation of hormone-dependent breast and prostate cancer cells and endothelial cells, and increased expression of genes associated with proliferation and angiogenesis [71]. PPAR*δ* can suppress the antiproliferative effects of PPAR*α* and PPAR*γ* [7] and directly associate with PDK1 [52] to affect its localization and activation [72, 73], which implicate it as a protumorigenic factor, and therefore raise a caution for the general use of this class of agonists [74].

**Figure 3:**
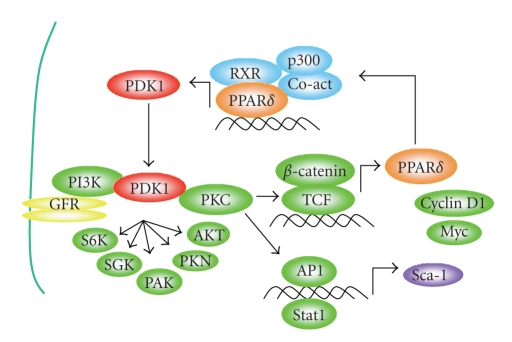
PDK1 and PPAR*δ* autoregulatory cascade. Growth factor receptor (GFR) activation activates PDK1 leading to PKC*α* and *β*-catenin/TCF activation [88]. TCF target genes include cyclin D1, c-Myc, and PPAR*δ* [75]. PPAR*δ* transactivates PDK1 [72], which in turn perpetuates the oncogenic signaling cascade. Preliminary data suggests that PDK1 maintains the expression of the murine stem/progenitor cell marker, stem cell antigen-1 (Sca-1), which is under the control of AP1 and Stat1 [92].

Colon cancer presents an interesting model to exam the role of PPAR*δ* in tumorigenesis since Apc^Min^ mice exhibit constitutive activation of *β*-catenin/TCF signaling, the pathway believed to activate PPAR*δ* [75]. PPAR*δ* is highly expressed in colorectal cancer cells [75], and somatic cell knockout of PPAR*δ* reduced tumorigenicity in nude mice [76]. Crossing PPAR*δ* null or heterozygous mice with Apc^Min^ mice showed a gene dosage dependent reduction in large intestinal polyps [65], and treatment of Apc^Min^ mice with GW501516 produced an increase in both polyp number and size [77], all suggesting that PPAR*δ* is protumorigenic. However, a study using a different targeting scheme to delete PPAR*δ* reported no change in polyp number or size in the small intestine of Apc^Min^ mice, and a greater number but not size of carcinogen-induced colon tumors in mice with this background [78]. Since the PPAR*δ* knockout mice generated by Barak contained a deletion of exon 4 encoding the hinge region [65], whereas, that generated by Peters et al. [64] contained a deletion of the last exon encoding the AF2 domain, it is possible that the truncated PPAR*δ* may not be as susceptible to corepression as the wild-type receptor. This would explain why their results [79, 80] differ from studies showing that keratinocytes from mice heterozygous or null for PPAR*δ* exhibit less proliferation [81] and those in Apc^Min^ mice in a PPAR null background exhibit increased tumorigenesis [65]. From a mechanistic standpoint, PPAR*δ* is activated in colon cancer cells by prostacyclin (PGI_2_) [82] and inhibited by the NSAID indomethacin [75], suggesting that its tumor promoting action is related to inflammation, a condition that increases the risk of colon cancer [83]. NSAIDs downregulate PPAR*δ* and reduce eicosanoid-mediated inflammation [84], and induce apoptosis in colon cancer cells [85], in contradistinction to the anti-inflammatory effects elicited by PPAR*γ* agonists in colitis [86]. Increased expression of PPAR*δ* in tumors may also inhibit PPAR*γ* transcription [60, 66, 67], and reduce its tumor suppressor activity, as mentioned above in colon tumorigenesis. In addition, the tumor promoting effects of PPAR*δ* in the mammary gland relate to activation of *β*-catenin/TCF signaling [76, 87] (see Figure 3), which is increased in cells transformed by PDK1 [88, 89]. PDK1 is a key regulator downstream of PI3K that is increased by PPAR*δ* in keratinocytes [72, 73]. Mammary tumors formed after administration of GW501516 exhibit an association between PDK1 and PPAR*δ* [52], which further suggests that PPAR*δ* may function as an integrator of proliferative and prosurvival pathways downstream of oncogenic signaling and inflammation [90, 91], which are likely to account for its tumor promoting effects.

**PPARs and stem cells**

There is evidence that PPARs can modulate stem and progenitor cell expansion and the differentiated or malignant phenotype. PPAR*γ* agonists enhance adipocyte differentiation [5, 6], and its ability to upregulate this process has a negative effect on osteoblast proliferation and bone development from mesenchymal stem cells [93]. To counteract this inhibitory effect in bone stem cells, PPAR*γ* must be transrepressed through corepressor recruitment by the NF*κ*B and Wnt-5a pathways [94]. It is therefore likely that PPARs influence the fate of other stem and progenitor cell populations in normal and malignant tissues. PPAR*γ* agonists have been used as chemopreventive agents [8] to delay mammary carcinogenesis [51, 52]. One aspect to their chemopreventive action may relate to their influence on specific cell lineages, as in mesenchymal stem cells. Carcinogens target stem cells rather than terminally differentiated cells [95, 96] as well as hormone-responsive lineages [97] during mammary carcinogenesis. Carcinogenesis is markedly attenuated in PR-null mice[98], and is accelerated by progestin treatment of wild-type mice [52, 99–101], where progestins are believed to stimulate the proliferation of stem or early progenitor cells that are intrinsically more susceptible to tumor initiation [102]. The ability of PPAR*γ* and PPAR*δ* agonists to modulate distinct cell lineages during mammary tumorigenesis [52] also suggests that they modulate a complex transcriptional network linked to cell fate [3, 5]. PPAR*δ* agonist GW501516 promoted the development of adenosquamous carcinomas with high expression of the stem cell markers CK19 and Notch1, as well as Proliferin, a growth factor that mediates many of the effects of the stem cell marker, Musashi1, in mammary cells [103]. PPAR*δ* is expressed in the crypt cells of the small intestine and negatively regulates Hedgehog signaling to block differentiation [104], a process that would be expected to promote transformation. PPAR*δ* expression lies downstream of *β*-catenin/TCF [75], and activation of this pathway increases expression of luminal epithelial and myoepithelial cells [102] as well as mammary tumor cells expressing the stem cell marker Sca-1 [105]. Thus, PPAR*δ* activation may promote expansion of a less differentiated lineage or stem cells that is intrinsically more susceptible to tumorigenesis. The association of Wnt activation with stem cell expansion, activation of *β*-catenin/TCF signaling by PDK1, the identification of PPAR*δ* as a *β*-catenin/TCF target gene and PDK1 as a PPAR*δ* responsive gene, as well as the modulation of Sca-1^+^ stem/progenitor cells by the Wnt pathway, all suggest a common mechanism for the tumor promoting action of PPAR*δ* agonists that may involve stem and progenitor cell proliferation (see Figure 3). This mechanism also suggests that the development of PPAR*δ* antagonists may have utility as cancer therapeutics

PPAR*γ* increases expression of the PPRE-dependent tumor suppressor genes PTEN [22] and BRCA1 [23], suggesting that their chemopreventive effects may be related to the ability of these suppressor genes to promote a more differentiated lineage. On the contrary, inactivation of BRCA1 [25] and PTEN [26–28] should increase stem cell proliferation, which is precisely the case. This effect is similar to what has been described for PPAR*δ* agonists in preventing differentiation and increasing stem cell abundance, and would be expected to complement their tumor promoting activity. Although studies examining the influence of PPARs on cell fate determination are just in their infancy, many of the studies cited imply that their opposing roles in tumorigenesis may be related to their ability to control the programming of specific cell lineages.

## 4. CONCLUSIONS

The ability of PPAR agonists to modulate the transcriptional activity of this class of nuclear receptors has generated an enormous interest in being able to pharmacologically manipulate entire sets of genes that can modulate metabolism, inflammation, transformation, differentiation and thus, tumorigenesis. Both genetic and pharmacological approaches to determining the function of PPAR*γ* and PPAR*δ* have yielded some inconsistencies, but that may be explained by the inherent deficiency of either approach. Gene targeting resulting in a truncated gene product may not necessarily recapitulate gene inactivation, and homozygous loss of gene expression can affect the developmental programming of various tissues that can impact directly or indirectly on the outcome of tumorigenesis in a particular organ. By the same token, pharmacological approaches are fraught with the structure-specific and class-specific side effects inherent in most drugs, which may be unrelated to their specific actions on the drug target. Nevertheless, the majority of studies in this field implicate PPAR*γ* activation as an antitumorigenic and prodifferentiation factor, in contrast to the protumorigenic and less differentiated phenotype resulting from PPAR*δ* activation. Although the latter characteristic will likely preclude the clinical development of PPAR*δ* agonists, it will be interesting to see the outcome of current clinical trials utilizing PPAR*γ* agonists as antitumor and chemotherapy modulating therapy.
